# Detection of mixed populations of wild-type and YMDD hepatitis B variants by pyrosequencing in acutely and chronically infected patients

**DOI:** 10.1186/1471-2180-12-96

**Published:** 2012-07-12

**Authors:** Francisco C A Mello, Bárbara V Lago, Lia L Lewis-Ximenez, Carlos A Fernandes, Selma A Gomes

**Affiliations:** 1 Laboratório de Virologia Molecular, Instituto Oswaldo Cruz, FIOCRUZ, Av. Brasil 4365, Rio de Janeiro, RJ, 21045-900, Brazil; 2 Laboratório de Referência Nacional para Hepatites Virais, Instituto Oswaldo Cruz, FIOCRUZ, Av. Brasil 4365, Rio de Janeiro, RJ, 21045-900, Brazil; 3 Laboratório Central de Saúde Pública Noel Nutels, Rua do Resende, 118, Rio de Janeiro, RJ, 20231-092, Brazil

## Abstract

****Background**:**

Lamivudine (LAM) is associated with the highest known rate of resistance mutations among nucleotide analogs used to treat chronic hepatitis B virus (HBV) infection. Despite this, LAM continues in widespread use, especially in combination therapies. The primary LAM resistance mutation (rtM204V/I) occurs in the YMDD motif of HBV polymerase. The aim of this study was to characterize Brazilian HBV isolates from acute and chronic cases by direct sequencing, and to identify HBV quasispecies in the YMDD motif using a pyrosequencing method capable of detecting single-nucleotide polymorphisms. HBV DNA from serum samples of 20 individuals with acute HBV infection and 44 with chronic infection undergoing antiviral therapies containing LAM were analyzed by direct sequencing and pyrosequencing methods.

****Results**:**

Phylogenic analyses of direct-sequenced isolates showed the expected genotypes (A, D and F) for the Brazilian population in both acute and chronic infections. However, within genotype A isolates, subgenotype A2 was more frequently detected in acute cases than in chronic cases (P = 0.012). As expected, none of the individuals with acute hepatitis B had LAM-resistant isolates as a dominant virus population, whether detected by direct sequencing or pyrosequencing. However, pyrosequencing analyses showed that 45% of isolates (9/20) had minor subpopulations (4-17%) of LAM-resistant isolates. Among chronic patients undergoing LAM treatment, YMDD mutants were frequently found as a dominant virus population. In cases where wild-type virus was the dominant population, subpopulations of YMDD variants were usually found, demonstrating the complexity of HBV quasispecies.

****Conclusions**:**

YMDD variants were frequently detected as a minor population in acute HBV infection. The occurrence of pre-existing variants may lead to a high frequency of resistant mutants during antiviral therapy in the chronic phase. In chronic infection, detection of YMDD variants before virological or biochemical breakthrough might contribute to making better therapy choices and thus improving treatment outcome.

## **Background**

The genetic variability of hepatitis B virus (HBV) contributes to the development of drug resistance, the major drawback of currently used antiviral treatments for chronic hepatitis B. Nucleoside/nucleotide analogs (NAs) are orally administered drugs designed to inhibit the function of HBV reverse transcriptase (rt). Although these drugs are highly effective in controlling viral replication, their efficacy is often hindered by the selection of drug-resistant viruses [1]. The selection pressure imposed by the presence of the drug gradually favors an increase in the population of viruses with mutations that confer resistance to the drug; this is often followed by an increase in viral load and serum alanine aminotransferase levels, and progression of liver disease [2,3]. Lamivudine (LAM), the first NA drug licensed for the treatment of chronic hepatitis B, is currently still in widespread use. This continuing use is in addition to more recently developed drugs that are efficient in the rescue therapy of LAM-resistant mutant [4]. LAM use is associated with the highest rate of resistance among NA drugs; this resistance progressively increases over the course of treatment, ultimately affecting 80% of patients after 48 months of administration [5-7]. The main site within the HBV rt protein that is associated with LAM resistance is residue 204 in the highly conserved tyrosine-methionine-aspartate-aspartate (YMDD) motif of the nucleotide-binding site; in general, the methionine in this sequence is replaced by either valine or isoleucine (rtM204V/I) [8,9]. This primary LAM-resistant mutant, rtM204V/I, affects viral replication fitness. Compensatory mutations in the rt domain (rtL180M, rtV173L, rtL80I/V) that partially restore replication efficiency are often co-selected in HBV rt204 mutants [1]. To date, the most commonly used method for detecting drug resistance mutations is by direct sequencing after polymerase chain reaction (PCR) amplification. In addition to being a laborious and time-consuming method, direct PCR sequencing is limited by its inability to detect variants that are poorly represented in the hete-rogeneous virus population present in a patient’s circulation. Therefore, other molecular techniques, including restriction fragment length polymorphism (RFLP) analysis [10-12], 5’-nuclease assays [10], melting point analysis [13], hybridization-based genotyping methods (e.g., mass spectrometry) [14], line-probe assays [15], DNA chip technology [16] and real-time PCR using mutation-specific primers [17], have been used to discriminate population mixtures [18,19].

Pyrosequencing is a new sequencing method that detects DNA polymerase activity by measuring the pyrophosphate (PPi) released by the addition of a dNMP to the 3’ end of a primer. It allows determination of the sequence of a single DNA strand by synthesizing a complementary strand, one base pair at a time, and detecting which base is added at each step. Pyrosequencing is currently the fastest, and probably most sensitive, method available for detecting small subpopulations of resistant virus and the unique capable of presents quantitative sequence data [7,19,20]. Here, HBV isolates from Brazilian patients with acute and chronic infections undergoing antiviral therapies containing LAM were genotyped and characterized by direct sequencing. Single-nucleotide polymorphisms (SNPs) in the YMDD motif of these HBV isolates were analyzed and quantified using a pyrosequencing method capable of rapidly sequencing short DNA sequences. Pyrosequencing results were compared with those obtained by direct sequencing.

## **Methods**

### **Serum samples**

In a parallel study [21], 129 samples from chronically HBV-infected patients undergoing interferon or NA analog therapy were examined for drug-resistance mutations. In the present study, serum samples from 44 of these patients undergoing LAM therapy as a first-line treatment were analyzed. These samples were referred to the public central Noel Nutels laboratory in Rio de Janeiro, Brazil, for the assessment of HBV loads. Individuals with clinical symptoms of acute hepatitis were monitored in the Viral Hepatitis Ambulatory Center of our Institution. The diagnosis of acute HBV infection was confirmed by positivity to anti-HBc IgM antibodies (AxSYM CORE-M; Abbott, Delkenheim, Germany). Twenty samples from these individuals were included in the present study. The research use of these samples was approved by the Fiocruz Ethics Committee, and written informed consent was obtained from all subjects.

### **HBV direct sequencing and HBV quantification by real-time PCR**

HBV DNA was extracted from serum samples using the High Pure Viral Nucleic Acid kit (Roche Applied Science, Mannheim, Germany) according to the manufacturer’s instructions. Viral DNA was eluted in 50 μL of Elution Buffer.

For the direct Sanger sequencing method, the pre-S/S genome region was amplified by semi-nested PCR. The first-round PCR product was amplified with the primer pair PS1 and P3, and the second round was performed using the sense primer PS1 and a mixture of two antisense primers, S2 and S22, as previously described [22]. DNA was amplified using 5 U/μL *Taq* DNA polymerase (Invitrogen, San Diego, CA, USA) and 10 mM dNTPs in a final volume of 50 μL. First round PCR was performed using the following conditions: 94°C for 3 min (initial denaturation), then 30 cycles of 94°C for 30 s, 55°C for 30 s and 72°C for 1 min 30 s, followed by a final elongation step (7 min at 72°C). Second-round thermocycling conditions were 94°C for 3 min, then 30 cycles of 95°C for 30 s, 52°C for 10 s and 72°C for 2 min, followed by a final elongation step (7 min at 72°C). The lower limit of detection of the PCR assay was 100 copies/mL. PCR products were purified using the Wizard SV Gel and PCR Clean-Up System (Promega, Madison, USA), and were prepared for sequencing using a Big Dye Terminator 3.1 Cycle Sequencing Kit (Applied Biosystems, Foster City, CA, USA) with external primers PS1 and S2 or S22, internal sense primer S4 (5'-TGCTGCTATGCCTCATCTTCT-3'; nucleotides [nt] 416-436) and antisense primer S7 (5'-TGAGCCAGGAGAAACGGGCT-3'; nt 676-656). The sequence was determined by separation and analysis of extension products using an automated ABI 3730 DNA Analyzer (Applied Biosystems). HBV genotyping was performed by phylogenetic analysis of the pre-S/S gene of the sequences determined in this study in the context of HBV sequences representing all known genotypes available in GenBank. Sequences were aligned using the ClustalW program [23], and the phylogenetic tree was generated using the neighbor-joining method (bootstrap resampling test with 1,000 replicates) in MEGA version 4.0 software [24]. Drug-resistance mutations were analyzed based on the deduced amino acid sequence of the region of the Pol open reading frame that overlaps the pre-S/S gene covering the rt domain from amino acid 1 to 230. Samples positive for HBV DNA were quantified by TaqMan real-time PCR technology, as previously described [25], using the probe, 5’-FAM-TGTTGACAARAATCCTCACAATACCRCAGA-TAMRA-3´ (nt 218-247). The assay has a limit of detection of 10 copies/reaction (i.e., 100 copies/mL serum).

Categorical variables were compared using Fisher’s exact tests, and differences between continuous variables were assessed using Student’s t-tests. Differences were considered statistically significant for *P*-values < 0.05. Statistical analyses were performed using SPSS version 17 (SPSS, Chicago, IL, USA).

### **Primer design and PCR assays for pyrosequencing**

Pyrosequencing was performed using PyroMark Q96 ID (QIAGEN Valencia, CA, USA). This instrument offers quantitative SNPs and mutation analysis by rapidly sequencing short stretches of DNA directly from PCR templates. PCR amplification and pyrosequencing primers were designed using PyroMark Assay Design 2.0 software. The following primers were designed to amplify a 218-bp fragment of the HBV rt polymerase domain containing the YMDD motif: forward primer, 5’-TTGCACCTGTATTCCCAT-3’ (nt 594-611); reverse primer, 5’-AAAATTGGTAACAGCGGTAWA AA-3’ (nt 791-812). The forward primer was 5’ biotin-labeled to enable preparation of a single-stranded template for pyrosequencing. The sequencing primer (5’-GTTTGGCTT TCAGYTAT-3’; nt 724-736) was located immediately upstream of codon rt204. DNA was amplified using 5 U/μL Platinum *Taq* DNA polymerase High Fidelity (Invitrogen), 10 mM dNTPs, 10X PCR buffer, 50 mM MgCl_2_ and 10 μM primer mix in a final volume of 50 μL under the following thermocycling conditions: initial denaturation at 94°C for 3 min, then 30 cycles of 94°C for 30 s, 55°C for 30 s and 68°C for 30 s, followed by a final elongation step (5 min at 68°C).

Biotinylated PCR products were hybridized to streptavidin-coated beads and purified using the PyroMark Q96 Vacuum Prep Workstation (Qiagen, Valencia, CA, USA) according to the manufacturer’s instructions. Sequencing primers were annealed by incubating at 80°C for 2 min. Pyrosequencing reactions were performed using the PyroMark Gold Q96 SQA Reagents in the PyroMark Q96 ID (QIAGEN). The dispensation order algorithm for pyrosequencing was CAGTACGCATG. Data collection and quantification analyses were performed using PyroMark ID software. Mixtures of plasmids carrying wild-type (WT) and YVDD-resistant (MUT) sequences were prepared to evaluate the ability of the pyrosequencing method to accurately detect and quantify minor sequence variants. Mixtures ranging from 100% WT-0% MUT to 0% WT-100% MUT were prepared at increments of 10% of each plasmid. A mixture of 95%-5% of each plasmid was tested to assess the sensitivity of the pyrosequencing assay in detecting minor subpopulations as low as 5% of the total.

## **Results and discussion**

Genotyping and subgenotyping of circulating HBV isolates in the samples analyzed (Table 1) showed no significant differences in genotype distributions among acute and chronic infections under LAM selective pressure. In acute infection, 16 of 20 HBV isolates (80%) under study belonged to genotype A, three (15%) were from genotype D, and the remaining one (5%) belonged to genotype F. In samples from chronic cases, the following genotype distribution was found: 25/44 (56.8%) genotype A, 13/44 (29.5%) genotype D, 5/44 (11.4%) genotype F, and one (2.3%) genotype B. Among isolates from genotype A, subgenotypes A1 and A2 were found. The ratio of subgenotypes A1/A2 in acute cases (8/8, 50% each) was significantly different from that in chronic cases (22/3, 88% A1 and 12% A2; *P* = 0.012). If the equal distribution of subgenotypes A1 and A2 among newly infected individuals (acute infection) reflects an increase in subgenotype A2 in Brazil, this suggests that the profile of circulating subgenotypes in Brazil could be changing. Alternatively, differences between the two subgenotypes could be related to disease progression (resolution of acute infection or progression to chroni-city). These possibilities warrant further investigation.

**Table 1:** Comparisons of YMDD variants in serum of patients with acute and chronic HBV infection detected by direct sequencing and pyrosequencing

Patient number	Type of infection	Treatment	Duration (months)	Viral load (cp/mL)	Sub genotype	Direct sequencing	Pyrosequencing % amino acid			
						WT	MUT	M	V	I
						ATG	Codon	ATG	(codon)	(codon)/(codon)
1969	acute	-	-	1.1x10^6^	A1	M/ATG	-	100	-	-
2098	acute	-	-	1.4x10^6^	A1	M/ATG	-	100	-	-
1377	acute	-	-	3.5x10^4^	A1	M/ATG	-	100	-	-
1504	acute	-	-	6.2x10^2^	A1	M/ATG	-	100	-	-
1379	acute	-	-	2.8x10^4^	A1	M/ATG	-	95	-	5 (ATT)
1419	acute	-	-	6.5x10^3^	A1	M/ATG	-	100	-	-
1781	acute	-	-	6.6x10^2^	A1	M/ATG	-	95	-	5 (ATT)
1510	acute	-	-	8.6x10^3^	A1	M/ATG	-	83	-	17 (ATA)
1384	acute	-	-	3.3x10^5^	A2	M/ATG	-	100	-	-
2190	acute	-	-	-	A2	M/ATG	-	94	6 (GTT)	-
603	acute	-	-	1.2x10^5^	A2	M/ATG	-	100	-	-
1472	acute	-	-	3.0x10^3^	A2	M/ATG	-	100	-	-
1386	acute	-	-	1.3x10^5^	A2	M/ATG	-	96	-	4 (ATT)
1120	acute	-	-	7.1x10^2^	A2	M/ATG	-	93	-	7 (ATT)
1393	acute	-	-	7.2x10^3^	A2	M/ATG	-	100	-	-
1889	acute	-	-	5.6x10^5^	A2	M/ATG	-	96	-	4 (ATT)
1474	acute	-	-	5x10^4^	D2	M/ATG	-	100	-	-
1980	acute	-	-	2.5x10^3^	D2	M/ATG	-	94	6 (GTT)	-
1314	acute	-	-	2.0x10^4^	D3	M/ATG	-	100	-	-
1570	acute	-	-	1.3x10^3^	F2	M/ATG	-	94	-	6 (ATT)
NN003	chronic	LAM	01	3.7x10^4^	A1	M/ATG		94	-	6 (ATT)
NN004	chronic	LAM	06	1.7 x10^4^	A1	M/ATG		96	-	4 (ATT)
NN124	chronic	LAM	06	9.7 x10^2^	A1	-	V/GTG	-	40 (GTG)	60 (ATT)
NN092	chronic	LAM	07	7.6 x10^6^	A1	M/ATG	-	100	-	-
NN006	chronic	LAM + TDF	12	1.7 x10^4^	A1	-	V/GTG	-	100 (GTG)	-
NN026	chronic	LAM	12	1.2 x10^7^	A1	-	V/GTG	-	100 (GTG)	-
NN041	chronic	LAM	12	1.3 x10^4^	A1	M/ATG	-	94	-	6 (ATT)
NN043	chronic	LAM	12	4.2 x10^5^	A1	M/ATG	-	100	-	-
NN132	chronic	LAM	12	9.4 x10^2^	A1	-	V/GTG	10	75 (GTG)	15 (ATT)
NN123	chronic	LAM	18	2.4 x10^9^	A1	M/ATG	-	94	-	6 (ATA)
NN009	chronic	LAM	24	2.1 x10^4^	A1	-	V/GTG	-	100 (GTG)	-
NN024	chronic	LAM	24	5.2 x10^7^	A1	-	V/GTG	-	100 (GTG)	-
NN010	chronic	LAM	36	4.9 x10^5^	A1	-	I/ATT	3	-	93 (ATT)/4 (ATA)
NN018	chronic	LAM	36	4.6 x10^3^	A1	-	V/GTG	6	94 (GTG)	-
NN019	chronic	LAM	36	3.0 x10^3^	A1	M/ATG	-	96	-	4 (ATA)
NN027	chronic	LAM	36	2.8 x10^4^	A1	M/ATG	-	95	-	5 (ATT)
NN037	chronic	LAM	36	4.8 x10^5^	A1	M/ATG	-	100	-	-
NN079	chronic	LAM	36	9.6 x10^3^	A1	M/ATG	-	100	-	-
NN087	chronic	LAM	72	1.1 x10^4^	A1	M/ATG	-	100	-	-
NN007	chronic	LAM	84	2.8 x10^4^	A1	-	V/GTG	-	100 (GTG)	-
NN028	chronic	LAM	108	1.8 x10^9^	A1	V/GTG	-	100 (GTG)	-	
NN032	chronic	LAM + TDF	132	1.2 x10^4^	A1	-	V/GTG	-	100 (GTG)	-
NN025	chronic	LAM	05	4.3 x10^4^	A2	M/ATG	-	100	-	-
NN014	chronic	LAM	07	1.4 x10^5^	A2	-	I/ATT	-	-	100 (ATT)
NN042	chronic	LAM	12	5.4 x10^7^	A2	-	V/GTG	6	94 (GTG)	-
NN022	chronic	LAM + ADV	24	1.7 x10^5^	B	-	I/ATT	-	25 (GTG)	70 (ATT)
NN074	chronic	LAM	06	6.5 x10^5^	D2	-	V/GTG	-	100 (GTG)	-
NN125	chronic	LAM + TDF	12	2.5 x10^3^	D2	-	I/ATT	-	-	100 (ATT)
NN001	chronic	LAM	60	2.4x10^4^	D3	-	V/GTG	-	100 (GTG)	-
NN091	chronic	LAM	06	4.3 x10^3^	D3	-	I/ATT	-	-	100 (ATT)
NN096	chronic	LAM	06	3.1 x10^3^	D3	M/ATG	-	100	-	-
NN097	chronic	LAM	06	5.3 x10^6^	D3	M/ATG	-	95	-	5 (ATT)
NN129	chronic	LAM	06	7.2 x10^8^	D3	-	V/GTG	-	95 (GTG)	5 (ATT)
NN029	chronic	LAM	12	7.0 x10^4^	D3	M/ATG	-	86	4 (GTG)	6 (ATA)/4 (ATT)
NN038	chronic	LAM + TDF	12	2.9 x10^5^	D3	M/ATG	-	100	-	-
NN077	chronic	LAM	12	9.7 x10^5^	D3	-	I/ATT	4	-	96 (ATT)
NN034	chronic	LAM + ADV	24	1.0 x10^5^	D3	-	V/GTG	-	90 (GTG)	10 (ATT)
NN075	chronic	LAM	60	3.2 x10^3^	D3	M/ATG	-	100	-	-
NN031	chronic	LAM	72	6.8 x10^7^	D3	-	V/GTG	-	100 (GTG)	-
NN126	chronic	LAM	06	1.9 x10^8^	F1b	-	I/ATC	-	30 (GTG)	70 (ATC)
NN105	chronic	LAM	06	3.7 x10^8^	F2	-	V/GTG	-	100 (GTG)	-
NN078	chronic	LAM	12	1.2 x10^6^	F2	M/ATG	-	95	-	5 (ATT)
NN134	chronic	LAM	12	2.7 x10^4^	F2	-	I/ATT	-	25 (GTG)	75 (ATT)
NN020	chronic	LAM	48	3.7 x10^4^	F2	M/ATG	-	100	-	-

Surprisingly, acute HBV patients had relatively low HBV titers compared to what would have been expected for an acute HBV infection, ranging from 6.2 x 10^2^ to 1.4 x 10^6^ copies/mL (mean viral load, 2.0 x 10^5^ copies/mL; median viral load, 2.0 x 10^4^ copies/mL). Chronic patients had HBV titers ranging from 9.4 x 10^2^ to 2.4 x 10^9^ copies/mL. The mean viral load was 1.4 x 10^5^ copies/mL, and the median was 5.6 x 10^4^ copies/mL.

**Figure 1:**
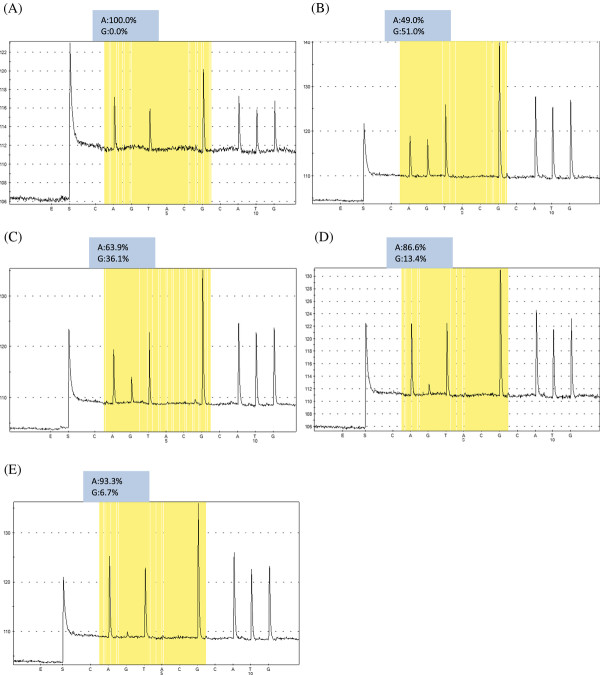
**Pyrosequencing analysis with allelic quantification of A/G for the first position of codon M/ATG and V/GTG in different mixtures of WT (YMDD) and MUT (YVDD) plasmids.** (**A**) 100% WT-0% MUT; (**B**) 50% WT-50% MUT; (**C**) 66% WT33% MUT; (**D**) 90% WT-10% MUT; (**E**) 95% WT-5% MUT. The results of quantification of each nucleotide are indicated above the pyrograms (as %).

The direct PCR Sanger sequencing method (population-based sequencing approach) detected only the major population in our assays. Literature reports indicate that only minor populations present as more than 20% of the total quasispecies pool can be detected by the Sanger method [26]. To test the ability of pyrosequencing to detect minor sequence variants of the YMDD population, we evaluated different proportions of plasmids containing WT (rtM204) and MUT (rtV204) sequences. Allelic quantification based on pyrograms indicated accurate detection when minor variants represented at least 5% of the total circula-ting population (Figure 1). A value of 4% was subsequently used as the lower limit of detection of minor populations by pyrosequencing under our experimental conditions. Other methods capable of detecting (but not capable of quantifying) viral mutants that constitute as little as 4-5% of the total population, such as RFLP analyses and line-probe assays, are either labor intensive and thus not suitable for high-throughput screening, or are subject to a greater number of false-positive and false-negative results than sequencing [7,20]. Our result confirms previous reports that pyrosequencing is the most sensitive method available for detecting small subpopulations of resistant virus and, as such, is likely to become the method of choice in the near future [7,19,20,27,28].

Comparisons of YMDD variants in serum of patients with acute and chronic HBV infection detected by direct sequencing and pyrosequencing are shown in Table 1. As expected, none of the individuals with acute hepatitis B had LAM-resistant isolates as a dominant virus population, whether detected by direct sequencing or pyrosequencing. However, because of its greater ability to detect viral subpopulations, pyrosequencing revealed that 11/20 (55%) of the individuals with acute hepatitis B had only WT isolates, whereas 9/20 (45%) had minor subpopulations of LAM-resistant isolates varying from 4% to 17%. The detection of pre-existing resistant variants in acute phase provides information helpful in choosing an appropriate antiviral regimen whether individuals have become chronic carriers, and thus need to start an antiviral regimen.

Thirty-eight patients (86.4%) with chronic hepatitis B were undergoing a LAM monotherapy regimen, whereas the other six (13.6%) were receiving combination therapy of LAM plus adefovir dipivoxil (ADV) or tenofovir disoproxil fumarate (TDF). There was no significant association between the treatment duration and the occurrence of LAM-resistant isolates. Direct sequencing methods determined that WT isolates were present in 19 of 44 patients (43.2%) and LAM-resistant isolates were present in 25 of 44 patients (56.8%), with a predominance of the YVDD variant (17/25, 68%) compared to the YIDD variant (8/25, 32%). Pyrosequencing confirmed the presence of exclusively WT isolates in 10 of 19 samples (52.6%) characterized as WT by direct sequencing. In the other nine samples (47.4%), pyrosequencing was able to detect the presence of minor subpopulations of LAM-resistant isolates. Of 25 samples characterized as LAM-resistant by direct sequencing, pyrosequencing confirmed the presence of only one population of resistant mutants (either YVDD or YIDD) in 14 (56%). The remaining 11 samples (44%) contained a mixture of mutant variant populations or a minor subpopulation composed of WT isolates. Pyrosequencing proved to be a powerful tool for detecting co-circulating strains in a complex population. This allowed resistant HBV to be detected before any evidence of virological or biochemical breakthrough, thus increasing the possibility of a correct choice of rescue therapy and increasing the likelihood of successful treatment.

**Figure 2:**
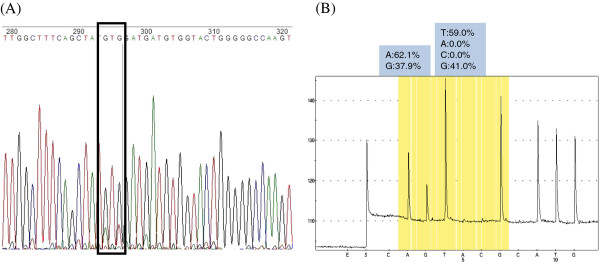
**Discrepancy between direct sequencing and pyrosequencing in sample NN124.** The direct sequencing method (**A**) detected the nucleotides (GTG) coding for the rtM204V variant, although the electropherogram indicated mixtures with small quantities of nucleotides A and T corresponding to the first and third nucleotide position of codon ATT (rt204I). Pyrosequencing (**B**) detected about 60% YIDD (I/ATT) and 40% YVDD (V/GTG) variants

Interestingly, all but two individuals whose major virus population was composed of WT isolates and a small percentage of resistant variants detected by pyrosequencing had a YIDD variant as a minor subpopulation, suggesting that the rtM204I mutation may naturally occur more often and replicate more efficiently than YVDD variants in environments with little or no selection pressures. The only disagreement between the results of direct sequencing and pyrosequencing was for sample NN124. The direct sequencing method detected nucleotides (GTG) coding for rt204V, although the electropherogram indicated mixtures with small quantities of nucleotides A and T corresponding to the first and third position, respectively, of codon rt204I (Figure 2A). In contrast, pyrosequencing indicated a majority (~60%) of rt204I variant and about 40% rt204V variant (Figure 2B). The same discrepant results were also obtained when the segment used as template for the direct sequencing method was amplified using pyrosequencing primers. This disagreement may be attributable to the similar amounts of YIDD and YVDD variants (60% vs. 40%) reported by pyrosequencing.

## **Conclusions**

Pyrosequencing is a rapid, specific, and sensitive tool that may be useful in detecting and quantifying subpopulations of resistant viruses. Here, YMDD variants were frequently detected by this method as a minor population in acute HBV infection. Co-circulation of mixtures of WT and mutant isolates of YMDD variants was frequently revealed in treated, chronic hepatitis patients by pyrosequencing. Detection of YMDD variants before their detection by conventional sequencing methods might contribute to making more informed drug choices and thus improving the outcome of therapy.

## **Authors' contributions**

FCAM and BVL carried out the sequencing experiments. LLLX was involved in the clinical evaluation of patients. CAF was responsible for demographic data of chronic patients. SAG conceived and coordinated the study. The manuscript was written by FCAM and SAG. All authors read and approved the final version of the manuscript.
